# Bio-collections in autism research

**DOI:** 10.1186/s13229-017-0154-8

**Published:** 2017-07-10

**Authors:** Jamie Reilly, Louise Gallagher, June L. Chen, Geraldine Leader, Sanbing Shen

**Affiliations:** 1 Regenerative Medicine Institute, School of Medicine, BioMedical Sciences Building, National University of Ireland (NUI), Galway, Ireland; 2 Trinity Translational Medicine Institute and Department of Psychiatry, Trinity Centre for Health Sciences, St. James Hospital Street, Dublin 8, Ireland; 3 Department of Special Education, Faculty of Education, East China Normal University, Shanghai, 200062 China; 4 Irish Centre for Autism and Neurodevelopmental Research (ICAN), Department of Psychology, National University of Ireland Galway, University Road, Galway, Ireland

## Abstract

Autism spectrum disorder (ASD) is a group of complex neurodevelopmental disorders with diverse clinical manifestations and symptoms. In the last 10 years, there have been significant advances in understanding the genetic basis for ASD, critically supported through the establishment of ASD bio-collections and application in research. Here, we summarise a selection of major ASD bio-collections and their associated findings. Collectively, these include mapping ASD candidate genes, assessing the nature and frequency of gene mutations and their association with ASD clinical subgroups, insights into related molecular pathways such as the synapses, chromatin remodelling, transcription and ASD-related brain regions. We also briefly review emerging studies on the use of induced pluripotent stem cells (iPSCs) to potentially model ASD in culture. These provide deeper insight into ASD progression during development and could generate human cell models for drug screening. Finally, we provide perspectives concerning the utilities of ASD bio-collections and limitations, and highlight considerations in setting up a new bio-collection for ASD research.

## Background

Autism spectrum disorder (ASD) is a group of early onset and heterogeneous neurodevelopmental disorders affecting males (1/42) more often than females (1/189) [1]. The prevalence of ASD has risen rapidly; from 0.5/1000 people in early epidemiological studies of 1960–1970 [2, 3] to 1/68 children of school age according to recent data from the Centre for Disease Control [1].

ASD is characterised by atypical development of social behaviour, communication deficits and the presence of repetitive and stereotyped behaviours [4]. It is highly clinically heterogeneous and accompanied by commonly occurring comorbidities that are not core to the disorder but frequently disabling. Communication deficit also persists in social communication disorder (SCD), and the new diagnosis of SCD (DSM-5) makes it possible to distinguish ASD from SCD individuals. The severity may vary across a range of parameters including ASD symptoms, IQ and comorbid behaviours [4]. For example, 70% ASD patients will have at least 1 comorbid psychiatric disorder [5], such as social anxiety, depression and bipolar disorder [6]. In addition, ASD is frequently associated with epilepsy, gastrointestinal and immune disorders [7].

ASD is a highly heritable complex polygenic condition. Estimated heritability based on family and twin studies are 50–80% [8, 9]. It is strongly linked to genetic factors involving the development and function of the nervous system [10], mitochondrial function [11], the immune system [12] and epigenetic regulations [13]. Genetic risk is attributed to rare copy number variants (CNV) and single nucleotide variants (SNV) acting on the background of common genetic variation (reviewed by [14]). High throughput genome sequencing technologies have facilitated genomic discovery, and advanced bioinformatics methodologies have enabled investigation of protein-protein interactions [15, 16] and functionally related pathways [17, 18]. The pathway to gene discovery has required large-scale international collaborative efforts based on the assembly of large bio-collections that are now publicly available and the subject of this review. In parallel to bio-collections, large-scale patient registries have provided epidemiological data that illustrate the course and prognosis of ASD and are helping to identify environmental factors influencing the aetiology [19–22]. Despite the advances, significant gaps in our knowledge of the aetiology remain and effective treatments for core ASD symptoms are elusive. The genetic and clinical heterogeneity of ASD means that further advancement will require larger bio-collections coupled with rich clinical data, ideally longitudinally to obtain a clear picture of the disorder both on the molecular and physiological levels.

### Autism bio-collections

A bio-collection is a large set of biologically characterised samples, such as blood or tissue collected from a group of individuals who typically have a specific medical condition. Bio-collections are useful as a dedicated resource to generate clinical and scientific data for the analysis of medical conditions on a large scale [23], as well as to create functional disease models to explore the biology of clinical conditions. Large-scale bio-collections and associated comprehensive data that can aid the interrogation of the relationship between the genotype and phenotype effects at the individual and group levels can address the issue of heterogeneity. The purpose of this review is to provide a summary of the publicly available ASD bio-collections, to highlight the impact of these on ASD research and to identify new directions for ASD bio-collection for future research purposes.

## Methods and search criteria

A literature search was conducted amongst published studies from Jan 2001 to Nov 2016 on electronic databases of Web of Science, EBSCO, PubMed, Science Direct, MEDLINE, Wiley Online Library. The search terms included “biobank”, “registry”, “collection”, “autism” and citation of bio-collections. A total of 263 studies from ASD bio-collections have been included in the tables and references of this review (Tables 2, 3, 4, 5 and 6).

### Inclusion criteria

This review included (a) studies using original samples of human tissues in ASD bio-collections; (b) studies using bio-samples extracted from systematically collected bio-resources (i.e. DNA, RNA, protein) for investigating the risk or influence of ASD; (c) the population studies involving participants of autism, Asperger and pervasive developmental disorder not otherwise specified (PDD-NOS); (d) studies published in peer-reviewed journals and (e) in English.

### Exclusion criteria

Studies were excluded (**a**) if they did not mention the collection(s) in the research data, references, acknowledgements or supplementary materials; (**b**) if the bio-samples were not derived from a systematic sample collection; and (**d**) if studies only concerned animal models of ASD without using ASD bio-collections or data.

We focus largely on studies from five bio-collections, four providing DNA, cell lines and metabolites, the Autism Genetic Resource Exchange (AGRE), Simons Simplex Collection (SSC), The Danish Newborn Screening Biobank (DNSB) and The Autism Simplex Collection (TASC) one providing brain tissue, Autism BrainNet (formerly the Autism Tissue Program (ATP)).We also included two emerging bio-collections that have fewer or no publications released yet, but could be of significant impact in the future. They are the Autism Inpatient Collection (AIC) [24] and the Autism Spectrum Stem Cell Resource [25]. An overview of the bio-collections and their website links can be found in Table 1.

**Table 1:** Information on the biobanks covered in the review

Name	Founded	Location	Type of sample collected and stored	Website/source paper
Autism Genetic Resource Exchange (AGRE)	1997	USA	Blood and immortalised cell lines	AGRE, www.agre.org
Simons Simplex Collection (SSC)	2010	USA	Blood and immortalised cell lines	https://sfari.org/resources/autism-cohorts/simons-simplex-collection
Danish Newborn Screening Bio-collection	1980’s	Denmark	Dried blood spot samples	http://www.ssi.dk/Diagnostik/Center%20for%20Neonatal%20Screening/Den%20Neonatale%20Screenings%20Biobank.aspx)
Autism Tissue Program	1998	USA	Post-mortem brain tissue	https://autismbrainnet.org/researchers/
Autism Spectrum Stem cell Resource	2014	USA	Skin fibroblasts, blood, induced pluripotent stem cells, neural stem cells, neuronal cells, glial cells	[25]
The Autism Simplex Collection	2010	USA and Europe	Blood	[97]
Autism Inpatient Collection	2014	USA	Blood and lymphoblasts	[24]

## Results

### Autism Genetic Resource Exchange (AGRE)

AGRE was established in 1997 by the Cure Autism Now (CAN) Foundation and the Human Biological Data Interchange (HBDI). Samples are provided by families with children affected by ASD and are coupled with anonymously coded clinical diagnostic data, such as Autism Diagnostic Interview–Revised (ADI–R) and Autism Diagnostic Observational Schedule (ADOS). Additional clinical data include photographic dysmorphology, neurological and physical examination, and family and medical history. AGRE is currently managed by Autism Speaks. It contains over 2500 families and the resource has contributed to high profile genetic discoveries relating to ASD (Table 2). Samples are housed at the National Institute of Mental Health repository at Rutgers’ University in the form of immortalised cell lines, DNA and serum samples which can be accessed by researchers through applications [20].

**Table 2:** Overview of studies using the AGRE collection

Reference	Bio-collection	Samples	Number	Study	Findings
[35]	AGRE	Genomic data (AGRE), brain tissue (mouse)	4327 samples (AGRE) 8789 samples (total)	Genotype-phenotype study	*HMGN1* found to be a negative regulator of *MECP2* expression. Dysregulation alters behaviour in mice, and AGRE collection contains genotypes linked to altered expression
[180]	AGRE	Blood	152 subjects	Quantitative trait analysis	Chromosome region 7q found to be a risk region for Autism Symptoms
[181]	AGRE	Lymphoblasts	1438 subjects	Association study	*CNTNAP2* identified as an ASD susceptibility gene
[182]	AGRE	Blood	1794 subjects	Linkage analysis	Chromosome 7q35 may harbour a gene that could contribute to variability in spoken language
[183]	AGRE	Genomic data	455 subjects	Pedigree study	Association found with chromosome X region Xp22.11-P21.2, where gene *IL1RAPL1* is located and also implicated in ASD
[184]	AGRE	Blood and lymphoblasts	252 families	Gene expression analysis and association	*ROBO1-4* found to be associated with ASD. Low expression levels of *ROBO1-2* found in ASD patients
[185]	AGRE	Blood and lymphoblasts	3211 subjects	Gene association study	Analysis of SNP polymorphisms in *PCDHA* suggest it as a potential candidate gene for ASD
[186]	AGRE and ATP	Lymphoblasts and brain tissue	3211 subjects (AGRE) 21 subjects (ATP)	Gene association study	*ZNF804A* found to be associated with ASD and verbal deficits, where knockdown of this gene reduced expression of *SNAP25*, and both are reduced in the anterior cingulate gyrus in ASD brains.
[187]	AGRE	Blood and lymphoblasts	72 families	Association study	Common variant found in *CNTNAP2* that is linked to ASD susceptibility
[43]	AGRE	Blood	470 families (total) 224(AGRE)	Association study	*GABRB3* and *GABRG3* found to be associated with ASD
[188]	AGRE	Blood and lymphoblasts	975 subjects	CNV analysis	Analysis of 15q13.1-3 region revealed *APBA2* as an ASD candidate gene
[189]	AGRE	Blood and lymphoblasts	1577 subjects (total) 1526 subjects (AGRE)	CNV analysis	*CNTNAP2* detected in ASD patients suggested to have a contribution to the disorder
[74]	AGRE	Lymphoblasts	6 subjects	Proof of principle	48 genes showed differential expression between patients and controls. Many genes involved in signalling, focal adhesion and metabolism
[190]	AGRE	Lymphoblasts	18 subjects* (controls provided by AGRE)	Profiling study	Altered levels of *UBE3A* (1.5–2 fold increase) expression found in ASD patients with 15q11-14 duplications. *APP* and *SUMO* found to be decreased, and are involved in apoptosis
[40]	AGRE	Blood and lymphoblasts	334 families	Reanalysis of data set using different analysis method	Association found in chromosome 1, which was previously overlooked. Further evidence that 17q11 is associated with ASD
[191]	AGRE	Genomic data	12 families	Method paper	Description of parent of origin method to detect mosaic chromosomal abnormalities.
[192]	AGRE	Blood and lymphoblasts	518 families	Replication study and functional study	The gene *EN2* suggested to act as ASD susceptibility locus, and mutations could alter brain development
[41]	AGRE	Blood and lymphoblasts	389 families (AGRE) 518 families (total)	Association study	Haplotypes found in ASD families found to affect regulation of *EN2* gene expression
[75]	AGRE	Blood and lymphoblasts	954 subjects	Gene-gene interaction study	Glutathione pathway is implicated in autism
[28]	AGRE	Blood and lymphoblasts	6056 subjects (TOTAL) 4444 subjects (AGRE)	GWAS	*UBE3A, NRXN1, BZRAP*, and *MDGA2* found to have disruptive CNVs amongst ASD patients, some only occurring once amongst patients
[83]	AGRE	Genomic data	830 subjects	Methods paper	Use of disease symptoms improves detection of linkage in genetic data. Useful when heterogeneity is involved
[38]	AGRE	Blood	18 subjects	Genotype-phenotype study	3 out of 18 patients with ASD and macrocephaly had mutations in PTEN gene. Considered as ASD gene to be explored
[39]	AGRE	Blood and lymphoblasts	88 subjects (total) 39 subjects (AGRE)	Mutation screening	De novo missense mutation found in one patient with ASD and macrocephaly.
[193]	AGRE	Blood and lymphoblasts	95 families	Gene linkage study	Chromosome region 2q suggested to contain an autism susceptibility gene
[53]	AGRE	Blood	88 families(total) 62 families (AGRE)	Linkage analysis	*GABRB3* polymorphism found to be associated with ASD
[194]	AGRE	Blood	115 families	Linkage analysis	Analysis carried out for a ASD family subset with obsessive compulsive behaviours (*n* = 35) found evidence of linkage to chromosome 1 and further evidence on chromosome 6 and 19
[82]	AGRE	Blood and lymphoblasts	279 subjects	Method paper	Multiplex ligation-dependent probe amplification shown to be effective at detecting microduplications and deletions
[50]	AGRE	Genomic data	748 subjects	Association study	*MET* variants associated with social and communication phenotypes amongst people ASD
[49]	AGRE	Blood and lymphoblasts	2712 subjects (total) 631 subjects (AGRE)	Association study	Multiple genes implicated in the MET pathway with ASD, such as *PLAUR* and *SERPINE1*
[48]	AGRE	Blood	743 families (total) 283(AGRE)	Association study	*MET* promoter variant that decreases expression found to be associated with ASD
[195]	AGRE	Blood and lymphoblasts	109 subjects	Replication study	Independent sample from the same cohort showed same linkage association to chromosome region 17q21
[196]	AGRE	Blood	480 families	Genetic score study	3 risk SNPs (*ATP2B2, PITX1, HOXA1*) had high reproducibility in males, 2 in females (*MARK1, ITGB3*), and 3 across both genders (*CTNAP2, JARID2, EN2*).
[197]	AGRE	Blood	381 subjects	Association study	Association between ASD in males and *ATP2B2*
[198]	AGRE	Blood	2569 subjects	Functional genomics study	Combining functional genomics and statistical analysis helped identify common variants in ASD
[199]	AGRE	Blood	2837 subjects	Association study	Rare haplotype affecting promoter of *DLX1* found to be associated with ASD. No common variants found for *DLX* genes and *GAD1*
[200]	AGRE	Blood	2261 subjects	GWAS	The chromosome regions Xp22.33/Yp11.31 suggested to harbour male specific variants for ASD
[201]	AGRE	Blood	1132 subjects	QTL analysis	Chromosome regions 16p12-13 and 8q23-24 linked to harbour genes contributing to deficits in non-verbal communication in autistic patients
[202]	AGRE	Blood	993 subjects	Association study	Glu27 allele of *ADRB2* gene suggested to confer increased risk of autism, with pregnancy related stressors having an increased effect
[203]	AGRE	Blood and brain tissue	90 subjects	Gene identification	Identification of the gene CORTBP2 from autism candidate region 7q31
[54]	AGRE	Blood	611 families	Association study	Reinforced evidence that GABRA4 and GABRB1 are implicated in ASD. Other ethnic groups found to have SNPs in these genes
[204]	AGRE	Blood	228 families (total) 38 (AGRE)	Association study	*HOXG1* polymorphism A218G found to be associated in increased head circumference amongst ASD patients
[205]	AGRE	Genomic data	2165 subjects + 1165 families (total) 2165 subjects (AGRE)	GWAS	Associations found in the following genes with ASD and linked co-morbidities; *KCND2*, *NOS2A* and *NELL1*
[206]	AGRE	DNA	37 twin sets (total) 15 twin sets (AGRE)	Association study	Terbutaline exposure for two or more weeks associated with increased concordance for ASD. 2 polymorphisms for *ADRB2* associated with ASD
[207]	AGRE	Blood	284 families (total) 38 families (AGRE)	Linkage/association study	Variants of *PON1* found to be associated with ASD families in North America, but not in Italian families
[208]	AGRE	Blood	38 subjects	CNV study	Microdeletions and duplications on chromosome regions 3p26.3, 6q24, 22q11.2, 4q34.2 and 1q24 linked to ASD with physical anomalies. Genes *STXBP5* and *LRRN1* identified as candidate genes
[209]	AGRE SSC	Genomic data	2294 subjects (SSC) 579 subjects (AGRE) 35663	CNV analysis	Exploration of evolution of human specific *SRGAP2* genes. Rare duplications observed in SSC cohort for *SRGAP2C*.
[210]	AGRE	Genomic data from [211]	121 families	QTL-analysis	2 loci were identified in chromosomes 11 and 17 associated with social responsiveness in ASD families
[81]	AGRE	Blood	411 families (total) 371 families (AGRE)	Method paper	Detection of amplicons using mismatch repair. More amplicon variants were found in patients compared to controls
[212]	AGRE	Blood	66 subjects	Metabolite analysis	ASD families have lower levels of unprocessed Reelin protein in blood than controls
[213]	AGRE	Blood	90 subjects	Gene characterisation	*CADPS* and *CADPS2* characterised and cloned. Found to be activators of protein secretion. No disease specific variants found amongst ASD patients
[214]	AGRE	Genomic data	1146 subjects	Linkage analysis	Linkage peaks found for language—speech phenotypes consistent with potential motor speed disorder in following chromosome regions; 1q24.2, 3q25.31, 4q22.3, 5p12, 5q33.1, 17p12, 17q11.2, 17q22, 4p15.2 and 21q22.2. multiple candidate genes were also identified
[215]	AGRE	Blood	2140 subjects	Linkage analysis	Parental origin effect significantly linked to chromosomes 4, 15 and 20
[42]	AGRE	Blood	167 families	Association study	*EN2* found to be associated with ASD susceptibly
[216]	AGRE	Blood and lymphoblasts	537 subjects (total) 34 subjects (AGRE)	CNV analysis	Proposal that increased CNV load, particularly duplication of base pairs, predisposes to ASD. Negative correlation found with CNV load and social and communication skills. Applied to both common and rare CNVs
[73]	AGRE	Blood and lymphoblasts	4714 subjects (total) 1336 subjects (AGRE)	CNV analysis	Genes involved in Neuronal adhesion (*NLGN1, ASTN2*) and ubiquitin pathways (*UBE3, PARK2, RFWD2, FBXO40*) were found in ASD patients. Further evidence of *NRXN1* and *CNTN4* involved with ASD.
[217]	AGRE	Blood	147 subjects	Genotype phenotype	Suggested relationship between polymorphism *MTFR* 677C → T and autism-related behaviours
[218]	AGRE	Blood and lymphoblasts	693 subjects (AGRE) 5878 subjects (total)	CNV analysis	Microduplications and microdeletions in chromosome 16p11.2 associated with psychiatric disorders; duplications associated with schizophrenia, bipolar disorder and ASD, and deletions with ASD and other neurodevelopmental disorders
[219]	AGRE	Blood	219 subjects	Variant analysis	*DLX1/2* and *DLX5/6* gene analysis may not contribute to ASD but functional analysis of variants still worth investigation
[36]	AGRE	Blood	1410 (total) 401 (AGRE)	Association study	No association found for a sequence variant in mental retardation found in exon 1 of MECP gene in autism cohort
[220]	AGRE	Blood and lymphoblasts	112 families(total) 79 families (AGRE)	Association study	A haplotype for *DRD1* is found to be associated with ASD risk amongst males
[221]	AGRE	Data from [222]	551 subjects (AGRE)	SNP analysis	Analysis of SNPs revealed variants of *CD38* associated with ASD. Variants of *CD38* linked to control of OXT secretion.
[223]	AGRE	Lymphoblastoid cell	14 subjects	Gene expression analysis	First study to show differential expression between lymphoblastoid cell lines. Genes affected implicated in cell death and development, nervous system development and immune development and function
[224]	AGRE	Lymphoblasts	116 subjects	Gene expression analysis	Patients with severe ASD showed altered expression of genes involved in Circadian rhythm. 20 novel genes found putative non-coding regions associated with androgen sensitivity
[225]	AGRE	Genomic data	1295 families (total) 696 families (AGRE)	GWAS	Noise reduction filter for GWAS leads to list of 830 candidate genes, where they impact dendrite and axon outgrowth and guidance
[29]	AGRE ATP	Blood and brain	133 sib pairs (total) 77 Sib pairs (AGRE) 17 brain tissue (ATP)	Ogliogenic hypothesis study	Evidence of epigenetic and genetic factors possibly contributing to ASD and *UBE3* having a possible role in ASD
[179]	AGRE	Blood and lymphoblasts	192 subjects (AGRE) 483 subjects (total)	Association study	Disruptions in *NRXN1* gene found to be associated with ASD
[226]	AGRE	Genomic data	476 subjects (total) 290 subjects (AGRE)	Association study	Suggestive association of parent and maternal origin effect on *SLC6A4* promoter variant and ASD. Further testing required on biological model or larger cohort
[26]	AGRE	Blood and lymphoblasts	1549 subjects 410 subjects (AGRE)	Mutation screening	Recurrent microdeletions in chromosome region 16p11.2 were observed in ASD patients and not in controls
[227]	AGRE	Blood and lymphoblasts	974 subjects (total) 512 subjects (AGRE)	Mutation screening	*RIMS3* identified as a possible ASD susceptibility gene
[228]	AGRE	Blood	33 families (AGRE) 49 families (total)	Association study	Association found for *HLA-DR4* gene in higher frequency in geographically defined subtype, but not in controls or AGRE sample
[229]	AGRE	Blood	508 families (total) 139 families (AGRE)	Association study	Analysis of 2p15-16.1 microdeletions region identified two candidate genes; *XPO1* and *OXT1*
[230]	AGRE	Blood and lymphoblasts	407 families (total) 138 families	Association analysis	Polymorphisms found in or near *DLX1* and *DLX2* found to be associated with ASD
[231]	AGRE	Blood and lymphoblasts	512 families (total) 138 families (AGRE)	Association study	Association found between ASD and *MTHFR* gene in simplex families but not in multiplex families
[37]	AGRE	Blood and lymphoblasts	219 families (total) 98 families (AGRE)	Association study	Polymorphisms in *MECP2* found to be associated with ASD
[232]	AGRE	Genomic data	990 families	Association study	2 genes found to be associated with ASD; *RYR2* and *UPP2*
[233]	AGRE	Genomic data	2194 families (total) 543 families (AGRE)	Association study	Association found between the calcium channel genes (*CACNA1L*, *CACNA1C* and *CACNA1*) with ASD
[32]	AGRE	Blood	470 families (total) 224 families (AGRE)	Gene association studies	*GABRA4* and *GABRB1* found to be associated with ASD
[234]	AGRE	Genomic data	680 families (AGRE) 1167 families (total)	GWAS	Identification of a common novel risk locus as chromosome region 5p14.1. Common and rare variants identified. AGRE used as validation dataset
[75]	AGRE	Lymphoblasts	12 subjects	Cell necrosis	Cells from autistic patients were more susceptible to oxidative and nitrosative stress
[235]	AGRE	Blood and lymphoblasts	1142 subjects (total) 139 families (AGRE)	Association study	*GTF2i* found to be associated with ASD
[236]	AGRE	Blood and lymphoblasts	207 families	CNV analysis	Translocation between short arms of chromosome 16 and 15 reported in 1 female patient. Nominal association of *A2BP1/FOX1* observed in ASD cohort.
[237]	AGRE	Serum	21 human subjects 13 rhesus monkeys	Exposure study	Monkeys exposed to antibodies from human mothers of autistic children displayed stereotypies and hyperactive behaviour. Autoimmune component suggested to contribute to ASD
[238]	AGRE ATP	Blood, lymphoblasts and brain tissue	276 families (AGRE) 17 subjects (ATP)	Association study	*MARK1* gene found to be associated with ASD. Overexpression of gene also found in prefrontal cortex (BA46) but not cerebellum in human post-mortem tissue. Mouse model showed abnormalities in dendrites.
[55]	AGRE	Blood	123 families (total) 75 Families (AGRE)	Linkage disequilibrium study	Nominal evidence found for ASD risk alleles in GABA_a_ Receptor subunits
[52]	AGRE	Blood	137 families (total) 80 families (AGRE)	Linkage and association study	*SLC6A4* found not to be associated to rigid-compulsive subset of ASD patients.
[239]	AGRE	Blood	158 families (total)	Linkage analysis	Increased support that chromosome regions 19p13 and 17q11.2 harbour ASD susceptibility loci
[240]	AGRE	Blood and lymphoblasts	1336 subjects (AGRE) 1509 subjects (total)	CNV analysis	Large-scale survey of 15q24 microdeletion syndrome identifies atypical deletion that narrows critical region and (776 kb versus 1.75mb) and number of genes (15 versus 38) sequencing of genes recommended
[241]	AGRE	Genomic data	4278 subjects (total) 1518 subjects (AGRE)	Transmission disequilibrium testing	AGRE dataset found to have a genome wide signals at chromosome region 10q26.13 in both sexes and paternal signals in 6p21.1
[30]	AGRE	Blood and lymphoblasts	2886 subjects (total) 1441 subjects (AGRE)	CNV analysis	Microdeletions and duplications at chromosome region 15q13.2q13.3 found to be associated with ASD symptoms and other psychiatric disorders
[242]	AGRE	Blood and Lymphoblasts	34 subjects	Linkage analysis study	Chromosomes 7q and 21q are associated with a subset of ASD patients with developmental regression
[222]	AGRE	Blood and brain tissue	1221 subjects (total) 263 subjects (AGRE)	Association study	Two genetic variants of *CD38* found to be associated with ASD
[243]		Blood	233 subjects	Association study	HOXA1 A218G alleles found to significantly influence head growth rates.
[244]	AGRE	Blood	196 families	Association study	Association not found between SNPs in *DLX6* and *PLCO* on chromosome 7q21-22 and ASD
[245]	AGRE	Blood	196 families	Association study	Presence of a susceptibility mutation found in *TDO2* or nearby gene
[246]	AGRE	Blood and lymphoblasts	249 families	Association study	Elevated levels of *STX1A* found to be associated with ASD
[47]	AGRE and ATP	Lymphoblasts	14 subjects (AGRE) 84 subjects (ATP)	Methylation study	Different methylation patterns found for genes involved in cell death/survival, neurodevelopment and gene transcription. Decreased expression of *RORA* and *BCL2* was found in brain samples of ASD patients
[247]	AGRE	Blood and lymphoblasts	110 subjects	Genetic association study	Association found between *PER1* and *NPAS2* and ASD
[248]	AGRE	Blood and lymphoblasts	104 families	Genetic association study	*BDNF* associated with ASD; significantly higher expression in ASD subjects
[249]	AGRE	Blood and lymphoblasts	13,205 subjects (total) 80 subjects (AGRE)	CNV analysis	Disruption of the *PTCHD1* locus on Xp22.11 identified in families with ASD and in families with Intellectual disability. Novel CNVs identified in *DPYD* and *DPP6*.
[80]	AGRE and ATP	Lymphoblasts and brain tissue	13 subjects (AGRE) 3 subjects (ATP)	Genotype-phenotype study	Increased dosage of the gene *CYFIP1* results in altered cellular and dendritic morphology and dysregulates mTOR pathway in ASD patients with duplications in 15q11-13
[250]	AGRE	Blood and lymphoblasts	95 subjects (AGRE) 134 subjects (total)	Genomic and molecular study	No coding mutations or parental-specific expression found in ASD and Gilles de la tourettes syndrome (GTS) in the gene *IMMP2L*. Gene should not be written out as factor for both conditions
[251]	AGRE	Blood and lymphoblasts	283 families	Linkage mapping study	*PRKCB1* shown to be associated with ASD
[252]	AGRE	Blood and lymphoblasts	1086 subjects	Candidate gene study	*PITX1* shown to be associated with ASD
[253]	AGRE	Blood	406 families (total) 99 Families (AGRE)	Association and linkage disequilibrium study	*GAD1* SNPs found not to be associated with ASD
[254]	AGRE	Blood	322 families (total) 86 families (AGRE)	Association study	No association found with *APOE* gene and ASD.
[255]	AGRE	Genomic data	4530 subjects	Association study	Immune function genes *CD99L2*, *JARID2* and *TPO* show association with ASD
[256]	AGRE	Blood and lymphoblasts	334 families	Association study	Analysis of 2q24-q33 region found following genes associated with ASD; *SLC25A12, STK39* and *ITGA4*
[257]	AGRE	Blood and lymphoblasts	411 families (total) 371 families (AGRE)	Linkage analysis	Linkage analysis of SNPs suggests *SLC25A12* to be associated with ASD
[258]	AGRE	Blood and lymphoblasts	352 families	Association study	No association found between polymorphisms in TPH1 and TPH2 and ASD susceptibility or endophenotypes
[259]	AGRE	Blood and lymphoblasts	352 families (total) 295 families (AGRE)	Association study	No association found between *SLC6A4* variants and susceptibility to ASD
[260]	AGRE	Blood and lymphoblasts	1011 subjects	Association study	*AHI1*, a gene associated with Joubert Syndrome, is also implicated in ASD
[261]	AGRE	Genomic data	2883 individuals	Methods paper	Tool that provides visualisation of SNP data
[262]	AGRE	Serum	34 subjects	Metabolite study	ASD patients had lower levels of the enzyme AAT in serum compared to controls. Difference is much more significant in ASD patients with regressive onset
[263]	AGRE	Blood and lymphoblasts	486 subjects (total) 252 subjects (AGRE)	Genotype-phenotype study	Mice with *CADPS2* knockout display autistic-like behaviour and cellular features. Analysis of human Cadps2 mRNA revealed aberrant splicing that resulted in some patients lacking exon 3 of the transcribed gene
[264]	AGRE	Blood and genomic data	860 subjects (total) 468 subjects (AGRE)	GWAS	Regions in 5q21.1 and 15q22.1-q22.2 found to have most significant association in combined data for Asperger. 8 regions overlap with ASD linkage areas, and 3 overlapped with a Finnish cohort
[79]	AGRE	Lymphoblasts	14 subjects	MicroRNA analysis	Dysregulation of MicroRNA expression contributes to gene expression in ASD. Gene targets *ID3* and *PLK2* were validated by knockdown and overexpression assays
[265]	AGRE	Genomic data	289 families	Method paper	SNPs involved in three-way epistatic interactions found and all located in gene GLRX3
[58]	AGRE	Blood and lymphoblasts	264 families	CNV analysis	De novo CNVs were found to be strongly associated with Autism
[266]	AGRE	Blood and lymphoblasts	248 subjects (total) 146 subjects (AGRE)	Association study	Results suggestive that a y-chromosome haplotype effect is associated with ASD
[267]	AGRE	Blood and lymphoblasts	196 families	Transmission analysis	Polymorphisms in *INPP1*, *PIK3G* and *TSC2* found to have linkage disequilibrium in ASD subjects
[268]	AGRE	Blood and lymphoblasts	196 families	Transmission analysis	Suggestive evidence that *GRM8* is a susceptibility gene in ASD
[269]	AGRE	Blood and lymphoblasts	196 families	Association study	Suggestive but tentative evidence for *MTF1* and *SLC11A3* as ASD suspectability genes
[270]	AGRE	Blood and lymphoblasts	10 subjects	Whole genome sequencing	59 candidate genes suggested to be associated with ASD susceptibility, with *ANK3* being the top result. 33 non-coding variants were also identified.
[271]	AGRE	Genomic data [73]	1336 subjects	Method paper	CNV analysis method that uses both B-allele frequency and log R ratio to find CNVs. Found all 21 validated short duplications in AGRE dataset. Analysis is much faster.
[272]	AGRE	Blood and lymphoblasts	Data taken from Ramoz, 2004	Association study	Suggestive association found for ASD-related routines and rituals with a polymorphism in *SLC25A12*
[273]	AGRE	Blood and lymphoblasts	144 subjects	Sequencing study	7 rare variants found in *NLGN3* and *NLGN4X*. UTR found not to be significant. 2 intronic variants suggested to influence regulation of genes. Limited by throughput and cost
[274]	AGRE	Blood	351 families	Association study	Nominal significance found for 15 genes, top 3 being *MYO1D*, *ACCN1* and *LASP1* suggested for further study
[275]	AGRE	Genomic data	148 families	Linkage analysis	Male-specific linkage mapped to chromosome 17q11. Evidence of sex specific risk alleles in ASD
[56]	AGRE	Lymphoblasts	284 subjects	Association study	*CACNAG* identified as a candidate gene for ASD
[276]	AGRE	Lymphoblasts	267 subjects (AGRE)	Linkage and association study	*SLC6A4* shown to contribute to ASD susceptibility
[78]	AGRE	Lymphoblasts	12 subjects	MicroRNA study	Lymphoblastoid cell lines from ASD patients can be used to assess microRNAs in ASD. Dysregulated MicroRNAs found to target genes linked to ASD
[277]	AGRE	Blood samples	100 subjects	Cholesterol metabolism	20% of the samples have shown hypercholestolemia, indicating that cholesterol metabolism could be perturbed in ASD
[278]	AGRE	Genomic DNA	756 subjects	Association study	*EGF* found to have significant association with ASD
[279]	AGRE and ATP	Data mining (AGRE) brain tissue (ATP) and blood	83 subjects	Linkage study	3p26.1, 3p26.3, 3q25-27 and 5p15 enriched for differentially expressed genes in blood and brain tissue. *CNTN4, CADPS2, SUMF1*, *SLC9A9* and *NTRK3* implicated in ASD and even more genes involved in neurological disorders that are co-morbid with ASD
[280]	AGRE	Blood	97 families	Expression profile analysis	*RAY1/ST7* locus found to contain a multi-transcript system. Screening of ASD patients found rare variants not present in controls
[281]	AGRE	Blood and lymphoblasts	196 families (total) 95 families (AGRE)	Mutation screening	No mutations found in coding regions of X-chromosomal *NLGN* genes.
[282]	AGRE	Blood and lymphoblasts	136 families (total) 96 families (AGRE)	Association study	High association of *FMR1* gene variant found amongst east Asian individuals, but not when whole sample was analysed, stratification confounded result
[283]	AGRE	Lymphoblasts	11 subjects	Neurotoxicity	Both ASD patients and controls showed upregulation of heat shock proteins when expressed to thimerosal
[33]	AGRE	Blood and lymphoblasts	3101 subjects (AGRE) 10796 subjects (total)	GWAS	Genome-wide SNPs found in *CDH10* and *CDH9* found to be associated with ASD
[284]	AGRE ATP	Blood, lymphoblasts and brain tissue	1031 families (AGRE) 3104 families (total) 30 subjects (ATP)	GWAS	Analysis found association in chromosome region 5p15, where genes *SEMA5A* and *TASR2* are located. Analysis of brain tissue showed reduced expression of *SEMA5A* in ASD subjects
[27]	AGRE	Lymphoblasts	5675 subjects (AGRE)	Association study	Micro deletion found in chromosome 16p11.2. amongst AGRE, Boston Children’s Hospital and Icelandic population data sets
[57]	AGRE	Blood	229 families	Association study	Sodium channel genes *SCN1A1-3* contained SNPs of interest amongst ASD families for future studies
[285]	AGRE	Blood	564 families (total) 327 families (AGRE) genetic analysis only 261 subjects (serotonin analysis)	Association study	*ITGB3* genetic variation found to be associated with serotonin blood levels and ASD susceptibility
[286]	AGRE	Genomic data	5328 subjects	Recurrence rate study	Significant difference in recurrence rates between male only families and female carriers in regard to ASD. Female protective effect suggested to be at work in high genetic-risk families involving female carriers. Shorter interbirth intervals correlated to ASD risk.
[287]	AGRE	Blood lymphoblasts	1587 subjects	Linkage analysis	Replication of linkage on 20p13. Linkage found for chromosomes 6q27, 8q13.2, 1p31.3, 8p21.2 and v8p12
[288]	AGRE	Lymphoblasts	75 subjects (total) 50 subjects (AGRE)	Gene characterisation	Gene characterised and assessed for mutation amongst ASD patients. No concrete association found
[289]	AGRE	Genomic data	487 families	Method paper	Pathways of interest analysed using GWAS SNP data. 5 pathways shown to be of significance in regards to ASD
[290]	AGRE	Blood and lymphoblasts	383 subjects	Loci analysis	AGRE and Finnish ASD dataset both showed strong association with 3p24-26 locus containing the gene *OXTR*
[211]	AGRE	Blood and lymphoblasts	833 families	Genome-wide screen	Evidence of linkage to ASD found on chromosomes 17, 5, 11, 4 and 8, of which 17 having the highest association score in the group
[291]	AGRE	Blood and lymphoblasts	110 families	Genome-wide linkage analysis	Nominal evidence for linkage found in chromosomes 2–4,8, 10–12,15-16,18 and 20. significant linkage found for chromosomes 5 and 8 after reanalysis
[292]	AGRE	Blood and lymphoblasts	389 families	Association study	No evidence found that RH -ABO foetal-maternal incompatibility is associated with ASD
[46]	AGRE	Blood	126 families (total) 81 families (AGRE)	Association study	*RELN* alleles with large CGG repeats may play a role in aetiology of certain ASD cases
[293]	AGRE	Blood and lymphoblasts	165 subjects	Population genetics	Study suggested two groups: low risk families caused by spontaneous mutations, and high risk caused by female offspring that carry ASD-causing mutation that is passed onto their own offspring
[294]	AGRE	Blood and lymphoblasts	205 families	Gene association study	No association found between ASD and variant of the gene *EN2*
[295]	AGRE	Lymphoblasts	20 subjects	Intracellular redox study	Inbalance of glutathione redox in cell lines derived from patients with ASD
[76]	AGRE	Lymphoblasts	86 subjects	Transmethylation/transsulfuration study	Cell lines derived from parents of ASD children showed abnormal transmethylation/transsulfuration metabolism and DNA hypomethylation

Study numbers listed as families or subjects wherever applicable

The AGRE resource has been used extensively in genomics studies in ASD. Approaches have included gene-mapping such as genome-wide linkage and association studies in addition to studies of chromosomal structure, particularly the identification of copy number variants. Important ASD chromosomal regions identified include microdeletions and microduplications of 16p11.2 [26, 27], rearrangements and microdeletion/duplication of 15q13.2q13.3 [28–31], common variants in the 5p14.1 region [32, 33], Neurexins and 11p12–p13 [34].

It has also helped in identification of recurrent candidate genes, such as *MECP2* [35–37], *PTEN* [38, 39], *EN2* [40–42], *RELN* [40, 43–46], *RORA* [47], *MET* [48–50], *NGLN3-4* [51], *BZRAP1* [28], *SLC6A4* [40, 52] GABA receptors [32, 43, 53–55], CACNA1G [56] and the sodium channel genes *SCN1A*, *SCN2A* and *SCN3A* [57].

These studies particularly highlighted an important role of de novo and large inherited copy number variations (CNVs), which are detected in 10% of sporadic ASD [58], which has been widely replicated in other bio-collections [59–71]. The use of AGRE combined with other AGP resources have uncovered *SHANK2*, *SYNGAP1*, *DLGAP2* and the X-linked *DDX53-PTCHD1* locus as novel ASD genes, as well as pathways of cellular proliferation, signalling, neuronal projection and motility [72]. AGRE samples formed a replication set in a separate analysis highlighting CNVs of neuronal cell adhesion and ubiquitin pathway in ASD [73].

AGRE lymphoblastoid cells enabled studies into shared ubiquitin and neuronal gene expression in lymphoblastoid cells and brain [73, 74], gluthathione metabolism, oxidative stress [75, 76] and stress response [77], microRNAs and their use in ASD profiling [78, 79], *CYFIP1* dosage effect on mTOR regulation [80], and changes in methylation patterns of *RORA* and *BLC2* and their effects on apoptosis, cellular differentiation, inflammation and neural development [47].

The AGRE collection was also used to establish genetic methodologies and bioinformatic tools. This included using mismatch repair to detect amplicons in ASD [81], using multiplex ligation-dependent probe amplification (MPLA) to improve detection of microduplications and microdeletions [82], and incorporating disease symptoms to improve linkage detection in genetic data [83] and analysis of genetic loci to search for candidate genes [84].

### Simons Simplex Collection (SSC)

The SSC is a genetic and clinical repository, which contains material derived from 2600 families. Whereas the AGRE contains multiplex families and trios, The SSC ascertained “simplex” ASD families defined as families where only one child has ASD and at least one other typically developing sibling. DNA is available for both parents, the affected child and an unaffected sibling. Thus the SSC samples are particularly valuable in evaluating parental inheritance. Samples were collected at multiple sites and were stored as immortalised cell lines at Rutgers University Cell and DNA Repository (RUCDR). Each sample was verified for parentage, gender and Fragile X mutation. In-depth clinical phenotypes were characterised for all participants to support genotype-phenotype analyses. These included data on diagnostic status, medical and psychiatric comorbidity, family history and medication use for the affected person. Broader ASD phenotype measures were collected for unaffected family members.

The SSC has become a vast resource of ASD and contributed significantly to numerous Whole exome sequencing studies of ASD in the past ~7 years (Table 3). The main findings showed that de novo mutations were frequently enriched in ASD patients [60]. Whole-genome sequencing results showed a significant enrichment of de novo and private disruptive mutations in putative regulatory regions of previously identified ASD risk genes. It also identified novel risk factors of *CANX*, *SAE1* and *PIK3CA* with small CNVs and exon-specific SNPs, which were overlooked in previous CNV studies or exon sequencing [85]. It has also been observed that many de novo mutations were of paternal origin (4:1) and positively correlated with paternal age, [65]. The disruptive mutations were located in genes involve in transcription regulation, chromatin remodelling and synapse formation [86, 87].

**Table 3:** Overview of studies using the SSC collection

Reference	Bio-collection	Samples	Number	Study	Findings
[296]	SSC	Genomic data	2760 subjects	CNV analysis	No association found between conception-assisted reproduction and risk of ASD
[297]	SSC	Lymphoblasts	900 subjects	Sequencing study	Rare functional variants of *TSC1/TSC2* did not show association with ASD
[298]	SSC	Genomic data	965 subjects (SSC)	Integrative analysis	Integrative analysis of data from 4 exome sequencing studies revealed enrichment of genes involved in chromatin remodelling and transcription in ASD patients
[88]	SSC	Blood	3730 subjects	Genotype-phenotype	Subtype of autism was caused by mutations to *CHD8*, of which 15 were found.
[299]	SSC	Blood	259 subjects	CNV analysis	Paired duplications mark cryptic inversions and other complex structural variations in CNV data.
[300]	SSC	Blood	552 subjects (total) 412 subjects (SSC)	Transcriptome analysis	Neuron development, nitric oxide signalling, neurogenesis and skeletal development were found outliers amongst ASD patients in TGEN cohort, whereas outliers were found in neurogenesis in ASD patients from SSC cohort
[301]	SSC	Blood and lymphoblasts	99 families	CNV analysis	55 potential pathogenic CNVs were identified and validated. 20% were considered rare when compared to the database of genomic variants. CNVs found in lymphoblast DNA but not in blood, suggesting pre-existing mutations may have been present in initial lymphoblast cells
[302]	SSC AGRE	Blood urine	12600 subjects (total) 1887 subjects (SSC) 752 subjects (AGRE)	Association study	*TMLHE* found to have high levels of deletion in male-male multiplex families (1 in 190) and deficiency of this gene could be a susceptibility factor for ASD.
[303]	SSC	Genomic and exomic data	Taken from earlier studies [60, 61, 70]	Genotype-phenotype study	Mutations in ASD candidate genes have greatest impact on pyramidal neurons, cortical neurons and medium spiny neurons. Truncating de novo mutations play a small role in high-functioning cases. The greater the functional disruption of genes, the more severe the phenotypes are.
[304]	SSC	Blood	2575 subjects	GWAS	Reducing phenotypic heterogeneity within the cohort did not have a significant effect on increasing genetic homogeneity.
[305]	SSC AGRE	Blood	14989 subjects (total) 5981 subjects (AGRE) 1815 subjects (SSC)	GWAS	CNVs found in *SEMA5* regulated gene network found to be associated with ASD
[86]	SSC	Blood	13,804 subjects	WES	104 genes were implicated in 5% of ASD cases, where they are involved in transcription, chromatin remodelling and synapse formation.
[59]	SSC	Blood	2963 subjects	WES	De novo INDELS primarily originate from father, frameshift INDELS associated with ASD, Frameshift INDELS more frequent in females. RIMS1 and KMT2E found to be associated with ASD
[306]	SSC	Blood	8 subjects	Methods Paper	WGS data more effective than WES for detection of INDELS. x60 sequencing required to recover 95% of detected Indels
[307]	SSC	Genomic data	2066 subjects	Homozygosity study	In ASD simplex families, increased runs of homozygosity is associated with Intellectual disability
[308]	SSC	Blood	1227 subjects (total) 350 subjects (SSC)	CNV analysis	CNV burden correlates to certain disorders; high CNV burden to Intellectual disability and low CNV burden to dyslexia
[178]	SSC AGRE	Blood	3168 subjects (total) 2478 subjects (SSC) 719 subjects (AGRE)	Rearrangement hotspot study	1q21 duplications found to be associated with Autism. CNVs identified in *CHD1L*, *ACACA*, *DPP10*, *PLCB1*, *TRPM1*, *NRXN1*, *FHIT* and HYDIN enriched in ASD. Duplications linked to decreased non-verbal IQ and duplications linked to severity of ASD.
[149]	SSC	IPSCs and lymphoblasts	1041 subjects	Disease modelling study	Disruption of *TRPC6* causes disruption in human neurons and linked to a non-syndromic form of ASD. First Study to use Patent-derived IPSCs to model non-syndromic form of ASD
[309]	SSC AGRE	Blood	2975 subjects (total) 1429 subjects (SSC) 14 subjects (AGRE	GWAS sequencing	Rare variants in synaptic genes associated with ASD. Loss of function in candidate genes a major risk factor for ASD.
[310]	SSC TASC	Blood	932 families (total)	Method paper	Transmission and de novo association(TADA) is a method that incorporates WES data, as well as inherited variants, and variants identified between cases and controls
[311]	SSC	Exome data	597 subjects	Method description	Association was found between ASD and rare variants of the gene *ABCA7* in exome data
[312]	SSC	Blood	15479 subjects (total) 9479 subjects (SSC)	Transmission analysis	Demonstration that high and low IQs could be distinguished by LGD load in respective gene targets. Transmission of rare variants with low LGD load occurs more often to affected offspring. Biased transmission towards children with low IQ
[61]	SSC	Blood	1478 subjects	WES	Gene disrupting mutations were twice as frequent in ASD subjects compared to controls. Genes disrupted were associated with Fragile X Protein FMRP.
[121]	SSC	Blood	762 subjects	CNV study	Female subjects showed a higher mutational burden before developing ASD.
[313]	SSC	Blood	720 subjects	Association study	Association was found between gene *SLC25A12* and restricted and repetitive behaviour.
[314]	SSC	Blood	2106 families (TOTAL) 965 families(SSC)	Common variation study	Multiple common variants of genes additively contribute to ASD risk. Simplex families found to closely follow additive model compared to multiplex families
[315]	SSC	Blood	285 subjects	Transcriptomic study	Enriched genes found in long term potentiation/depression, Notch signalling and neurogenesis amongst ASD Patients. 55 gene prediction model performed well on male subjects, but not female subjects
[316]	SSC	Blood	58 subjects	Transcriptomic study	Upregulation of spliceosome, mitochondrial and ribosomal pathways and downregulation of neuroreceptor-ligand, immune response and calcium signalling pathways in ASD patients compared to controls
[317]	SSC	Genomic data	78349 subjects (total) 3080 subjects (SSC)	SNP study	17–29% of variance in liability explained by SNPS. Genetic correlation found between disorders; High: Schizophrenia and bipolar disorder Moderate: Schizophrenia and major depressive disorder, major depressive disorder and ADHD, major depressive disorder and bipolar disorder Low: Schizophrenia and ASD
[60]	SSC	Genomic Data	1784 subjects	CNV study	De novo duplications and deletions are major contributors to ASD. Females shown to have a greater genetic resistance to autism.
[318]	SSC AGRE TASC	Blood	6970 subjects (total) 806 subjects (AGRE) 996 subjects (TASC) 563 subjects (AGRE)	WES	2-fold enrichment of complete knockout of autosomal genes with low LoF variation, and 1.5-fold enrichment for rare hemizygous knockout in males. Both contribute 3 and 2% to ASD risk, respectively.
[63]	SSC	Lymphoblasts	386 subjects	CNV study	Recurrent and rare de novo CNVs were discovered to alter gene expression in chromosome regions 3q27, 3p13, 3p26, 2p15, 16p11.2 and 7q11.23.
[129]	SSC	IPSCs	12 subjects	Disease modelling	Overexpression of *FOXG1* was linked to increased head circumference and ASD severity in idiopathic autism subjects. An overabundance of inhibitory neurons in ASD cell lines was also found.
[319]	SSC	Genomic and clinical data	2478 subjects	Gene-environment study	Individuals with ASD-associated CNVs were more susceptible to effects of febrile episodes and maternal infection during pregnancy and have impact on behavioural outcomes
[320]	SSC	Blood	10118 (TOTAL) 1974 (SSC)	Genetic association	Higher prevalence of *SLC12A5* variants containing altered CpG sites amongst ASD patients.
[321]	SSC DNSB	Blood	2418 subjects (SSC) 1353 subjects (DSNB)	CNV analysis	17q12 deletion identified as a CNV variant that confers high risk of ASD and Schizophrenia
[322]	SSC AGRE	Genomic data	49167 subjects (total) 1124 subjects (SSC) 1835 subjects (AGRE)	CNV analysis	More significant CNVs that could infer ASD risk were identified using combined large clinical datasets of neurodevelopmental disorders than with ASD cohorts alone
[323]	SSC	Lymphoblasts	5451 subjects	Association study	No association was found for heterozygous mutations in *CNTNAP2* and contribution to ASD risk
[324]	SSC	Blood and lymphoblastoid cell lines	593 families	Method description	A novel method was used to detect de novo and transmitted insert-deletions(Intel’s) in exomic data
[325]	SSC	Blood	1315 subjects (total) 145 subjects (SSC)	CNV analysis	Duplication CNVs enriched in negative regulation categories, deletion CNVs enriched in positive regulation categories. Highly connected genes in network enriched in patients with a single gene CNV change
[65]	SSC	Blood	677 subjects (SSC)	WES	De novo mutations paternal in origin (4:1) and positive correlation with age. Recurrent mutations in genes *CDH8* and *NTNG1*.
[64]	SSC	Blood	20 families (total) 19 families(SSC)	WES	21 de novo mutations identified. 11 of which found to be protein altering. Mutations identified in *FOXP1, GRIN2B, SCN1A, LAMC3* and *CNTNAP2.*
[87]	SSC	Blood	2246 subjects (SSC)	WES	27 de novo events found in 16 genes, 59% predicted to truncate proteins. further support for genotype-phenotype relationship in *CDH8* and *DYRKA1*
[326]	SSC	Blood	19 subjects (total) 4 subjects (SSC)	Genotype-phenotype	Overexpression/increased dosage of MECP2 related with core features of ASD
[133]	AGRE SSC	Data taken from [327]	8816 subjects (total) 737 subjects (SSC) 4449 (AGRE)	Replication study	Findings could not be replicated from Skafidas paper
[328]	SSC DNBS	Genomic data	38000 subjects (total) 4358 subjects (SSC) 19142 subjects (DNBS)	General population study	Genetic influences on ASD risk found to influence typical variation in social and communication ability in the general population
[67]	SSC	Blood	2256 subjects	De novo and familial influences	Familial influences were more significant in cases of high-functioning ASD conditions.
[327]	SSC	Lymphoblasts	1 subject	Clinical report	De novo microdeletion in chromosome 3q29 associated in person with ASD, childhood psychosis and intellectual disability
[68]	SSC	Genomic data	1024 families	De novo mutation analysis	Significant role for loss of function mutations in ASD cases.
[329]	AGRE SSC	Blood	8816 subjects (total) 737 subjects (SSC) 4449 subjects (AGRE)	Predictive testing	Diagnostic classifier containing 237 SNPs and 146 genes
[330]	SSC AGRE	Blood	975 subjects (total) 392 subjects (SSC) 585 subjects (AGRE)	Genotype-phenotype study	NPAS1 found to repress generation of specific subtypes of cortical interneurons
[85]	SSC	Blood	53 families	Whole genome sequencing	Enrichment of disruptive mutations in putative regulatory regions in ASD patients
[71]	SSC	Blood	9231 subjects	Genotype-phenotype study	Disrupting mutations in *DYRK1A* were linked to a subset of 15 patients with a syndromic form of ASD/ID.
[331]	SSC	Blood	903 families	WES	Enrichment of non-synonymous and potentially pathogenic mutations in mitochondrial DNA in ASD patients compared to controls. Transmission of potential pathogenic mutations differed between mother-ASD pairs and mother-sibling pairs
[332]	SSC	Lymphoblasts	1 family	Mutation analysis	PKA found to be an upstream regulator of *UBE3A*, where mutation in phosphorylation site results in hyperactivity of UBE3A
[333]	SSC	Blood	686 subjects + 612 families (SSC)	WES	Bi-allelic mutations found in genes enriched in inherited ASD cases (*AMT*, *PEX7*, *SYNE1*, *VPS13B*, *PAH*, *POMGNT1*)
[333]	SSC	Blood	928 subjects	WES	Strong evidence that de novo mutations are associated with ASD
[69]	SSC	Blood, lymphoblasts and saliva	1174 families	CNV analysis	Significant associations found between ASD and *de novo* duplications of chromosome 7q11.23. *de novo* CNVs identified in 5 other regions, including 16p13.2
[334]	SSC	Blood and lymphoblasts	2591 families	CNV analysis	De novo CNVs associated with ASD. 6 loci and 65 genes identified, many targeting the chromatin or synapse
[335]	SSC	Genomic data	2337 families	Transmission disequilibrium	Excess of truncating inherited mutations associated with ASD. *RIMS1, CUL7, LTZR1* identified as candidate genes
[336]	SSC	Genomic data	411 families	Transmission disequilibrium	Affected ASD patients inherited more CNVs than their unaffected siblings, and these CNVs of ASD patients affected more genes. Enrichment of brain-specific genes in inherited CNVs amongst ASD patients
[312]	SSC	Genomic data	10,942 subjects (total) 4942 subjects (SSC)	Biased transmission study	Frequent biased transmission of disruptive mutations to Low IQ ASD patients. Low and high IQ subjects can be distinguished by mutational load.

Study numbers listed as families or subjects wherever applicable

The SSC has enabled detection of the ultra-rare “recurrent” CNVs. This included duplications of 7q11.23, 15q11.2 (*NIPA*) and 16p13.11, and deletion/duplication of 16p11.2, 16p13.2 (*USP7*), 1q21.1, 2p16.3, 7q31.1, 15q13.2–q13.3, 16p13.3, 20q13.33 and 22q11.21 [60]. The SSC also helped identify recurrent gene mutations in ASD include *CHD8, NTNG1, GRIN2B, SCN1A* and *LAMC3*, which are important for transcriptional regulation, neuronal differentiation and function [87].

*CHD8* was further evaluated as an ASD candidate gene in children with developmental delay or ASD, and 15 independent mutations were identified and enriched in a subset of ASD with altered brain size, distinct facial features and gastrointestinal complaints. Disruption of *CDH8* in zebra fish recapitulated some of the patient phenotypes including increased head size and impaired gastrointestinal motility [88]. CHD8 is shown to control expression of other high-confidence de novo ASD risk genes such as *DYRK1A*, *GRIN2B* and *POGZ* [89]. Mutation of *DYRK1A* was strongly linked to a subset of ASD patients with seizures at infancy, hypertonia, intellectual disability, microencephaly, dysmorphic facial features and impaired speech [71, 89]. *POGZ* gene which plays a role in cell cycle progression is also found to contribute to a subset of ASD with varying developmental delay, vision problems, motor coordination impairment, tendency of obesity, microcephaly, hyperactivity and feeding problems [90].

### Danish Newborn Screening (NBS) Biobank

The NBS Biobank has a large collection of dried blood spot samples (DBSS), which are taken from new-borns 5–7 days after birth. They are sent to the New-born Screening lab at the Statens Serum Institute for analysis, and stored at −20 °C in a separate freezing facility at the NBS Biobank. Prior to collection, parents are informed via leaflets about the biobank, with focus on what the samples will be used for (documentation, testing and retesting, research, etc.). Participants can opt out of storage at any time via a letter to the department. For security, both the clinical data and biological samples are linked via a unique number, kept in separate buildings, and are accessible by authorised personnel only [91]. The advantage of the NBS resources is that it provides a large amount of non-ASD controls as well as Danish ASD samples.

In the past 30 years the NBS Bio-collection has accumulated samples from 2.2 million individuals, around 65,000–70,000 samples per year from Denmark, Greenland and the Faroe Islands. Most recently this resource has been included under the Danish iPsych consortium with the Psychiatric Genomics Consortium (PGC), added 8–12 k samples to the PGC analysis and significantly increased its power to detect common genetic effects for ASD, which have been recently published [92].

DBSS were also used to examine metabolites. A group led by Abdallah carried out a series of studies on Danish collections (Table 4) to examine the potential role of cytokines and chemokines involved in signalling and immune response of ASD. Initially using amniotic fluid from the Danish Birth Cohort (DBC) collection [93, 94], they followed up with DBSS from new-borns crossed referenced from that cohort [95, 96]; they detected an imbalance of cytokines amongst ASD subjects compared to the controls. Most of the chemicals were lower than normal, such as Th-1 and Th-2 like cytokines involved in proliferation, priming and activation of these cell types, whereas a small number of cytokines displayed increased expression in ASD. The abnormal levels of these chemicals could lead to a hypoactive or “inactive” immune system in the brain, making it more susceptible to infection-related ASD. However, when chemokine levels were examined in amniotic fluid, no concrete relationship could be established.

### The Autism Simplex Collection (TASC)

TASC is a trio-based international bio-collection that was assembled in collaboration with the Autism Genome Project and funded by Autism Speaks [97]. Trios, comprised of both parents and a child affected with ASD with no known medical or genetic cause. Collection of samples took place between 2008 and 2010 across 13 sites; 9 in North America and 4 in Europe. Management, storage and distribution of TASC data are handled by the Centre for Collaborative Genetic Studies on Mental Disorders (CCGSMD) [97]. Samples are housed at the NIMH and AGRE repositories both of which are located at Rutgers University.

So far, TASC has been used for GWAS studies [66] and CNV studies [72, 98, 99] and WES Studies [16, 100, 101]. In addition, TASC has also been used in WGS as part of the MSSNG project, which is discussed below

### Autism Inpatient Collection (AIC)

The AIC is a bio-collection for ASD research based on those on the serve end of the spectrum with severe language impairment, intellectual disability and self-injurious behaviour. This collection was founded on the basis that this segment of ASD patients are largely unrepresented in current studies. Bio-samples are initially recruited from 147 patients, and ongoing recruitment is estimated at 400 per year. Psychiatric, clinical and phenotypic data are collected in addition to blood samples for the creation of lymphoblastoid cell lines by RUCDR. Amongst this collection, over half are non-verbal, over 40% have intellectual disability and a quarter exhibit self-injurious behaviour [24]. This collection has yet to be used in any genetics-based studies. The fact that many patients are on the severe end of the spectrum makes it a welcome addition, and it opens opportunities to explore this under-represented group.

### Autism Tissue Program (ATP)/Autism BrainNet

The Autism Tissue Program, now the Autism Brain Network, is a post-mortem ASD brain collection coordinated by a network of parents, caregivers, physicians and pathologists. Brain samples are preserved in formalin and/or in −80 °C freezers to maximise the potential studies. In some cases, both hemispheres are fixed in formalin when there is freezing capacity or if the post-mortem interval exceeds 24 h. Corresponding clinical data include age, sex, ethnicity, diagnosis, brain size, cause of death, post-mortem interval and preservation method for the left and right hemisphere of the brain. Due to the rarity of the sample, a thorough application procedure assesses scope, scale and feasibility of proposed projects prior to access of tissue, with the expectation that data, images and presentations generated by research on the samples are provided back to the Autism Brain Network 3 months after formal release of publications [102].

Brain pathology and molecular mechanisms have been the focus of studies using the ATP resource (Table 5) although many studies looking at brain anatomy and cell morphology employed samples from this collection, molecular and genetic studies are the primary focus of this review. Such studies included transcriptomics [103–105], epigenetics [29, 106–115] and alternative splicing [116, 117]. A key discovery was the identification of convergent molecular pathology linking to neuronal, glial and immune genes [105] in a transcriptomics study that investigated the gene co-expression network between autistic and control brains. This led to the proposal of abnormal cortical patterning as an underlying mechanism due to attenuated differential expression in frontal and temporal cortices in ASD brains.

A recent study showed reduced Vitamin B12 in ASD brains [118] where the ATP made a very large contribution. Post-mortem examination of brain tissue ranging from foetal to the elderly subjects also showed a marked decline of the brain vitamin B12 with age, together with lower activity of methionine synthase in the elderly, but the differences were more pronounced in ASD and schizophrenia subjects when compared to controls. Acetylation is an important post-translational modification in the field of epigenetics. ATP also made a significant contribution to a large-scale histone acetylome-wide association study (HAWAS) using the prefrontal cortex, cerebellum and temporal cortex in ASD patients and controls. Despite their heterogeneity, 68% of syndromic and idiopathic ASD cases shared a common acetylome signature at >5000 cis-regulatory elements in the prefrontal cortex and temporal cortex. Aberrant acetylome was found to be associated with synaptic transmission, ion transport, epilepsy, behavioural abnormality, chemokinesis, histone deacetylation and immunity [113].

The ATP sample was used in a methylation study that investigated differential methylation in CpG loci in three brain regions: temporal cortex, dorsolateral prefrontal cortex and cerebellum. Differential methylation of four genes (*PRRT1*, *C11orf21*/*TSPAN32*, *ZFP57* and *SDHAP3*) was detected. *PRRT1*, *C11orf21*/*TSPAN32* were hypomethylated while the latter two were hypermethylated [109]. A further investigation in Brodmann’s area also found a pattern of hypomethylation of a number of genes including *C11orf21/TSPAN32* that are implicated in immune function and synaptic pruning [111]. These hypomethylated genes correlated with those showing overexpression by Voineagu.

The methylation studies have further uncovered dysregulation of *OXTR* and *SHANK3* genes in ASD. *OXTR* gene encoding oxytoxcin receptor was significantly hypermethylated in the peripheral blood cells and temporal cortex of ASD, highlighting a reduced oxytocin signalling in the aetiology of ASD [108] and a therapeutic target of ASD. Differential methylation of the *SHANK3* gene was detected between ASD and control brains. They found that when three 5′ CpG islands of the gene were examined, they observed altered methylation also changed *SHANK3* splicing, with specific *SHANK3* isoforms expressed in ASD [114].

This is echoed by a recent study, which reveals a dynamic microexon regulation associated with the remodelling of protein-interaction networks during neurogenesis. The neural microexons are frequently dysregulated in the brains of ASD, which is associated with reduced expression of *SRRM4* [116]. The neuronal-specific splicing factor A2BP1/FOX1 and A2BP1-dependent splicing of alternative exons are also dysregulated in ASD brain [105].

### Replication studies and pooling resources

Research data from one bio-collection is not always replicable in another sample set. Therefore, cross-validation between different bio-collections will not only minimise false positive, but also identify the common risk factors and subset-specific factors. For example, a genome-wide survey was carried out to test trans-generational effects of mother-child interactions, and the AGRE and SSC samples were used to replicate the original findings of 16 ASD risk genes (*PCDH9*, *FOXP1*, *GABRB3*, *NRXN1*, *RELN*, *MACROD2*, *FHIT*, *RORA*, *CNTN4*, *CNTNAP2*, *FAM135B*, *LAMA1*, *NFIA*, *NLGN4X*, *RAPGEF4* and *SDK1*) involving urea transport and neural development. The results from the AGRE and SSC cohorts did not match the original study and showed fewer associations. When post-correction of the statistics was applied, the results lost their significance [119]. This could partially be due to the differences in the array design with different coverage of SNPs and/or different methodologies.

The meta-analysis of five data sets including the AGRE and SSC demonstrates that females have a greater tolerance to CNV burden. This leads to a speculation that the maternal tolerance of the CNVs can result in decreased foetal loss amongst females compared to males, and that ASD-specific CNV burden contributes to high sibling occurrence. What is interesting about this study is that the results for high CNV burden in females are consistent throughout each data set. This is an example showing how multiple bio-collections can give a clearer picture in a combined study where individual studies may be ambiguous [120, 121].

Many major studies on the genetics of ASD have also been accomplished as a result of the collaborations amongst the institutions (Tables 2, 3, 4, 5 and 6). An effort was made to evaluate the association of Fragile X Mental Retardation 2 locus (*AFF2*) with ASD using joint resources from AGRE (127 males) and SSC (75 males). *AFF2* encodes an RNA-binding protein, which is silenced in Fragile X. The study found that 2.5% of ASD males carry highly conserved missense mutations on *AFF2* gene which was significantly enriched in ASD patients, when compared to >5000 unaffected controls [122]. A WES was published recently, which sequenced the exomes of over 20,000 individuals, including those from the SSC and Swedish registries. The study identified 107 candidate genes, and reinforced ASD pathways of synaptic formation, chromatin remodelling and gene transcription. This study detected mutations in genes involved in calcium- (*CACNA2D3*, *CACNA1D*) and sodium-gated channels (*SCN2A*) which were related to neuronal function, and in genes involved in post-translational methylation (*SUV420H1*, *KMT2C*, *ASH1L*, *SETD5*, *WHSC1*) and demethylation (*KDM4B*, *KDM3A*, *KDM5B*, *KDM6B*) of lysine residues on histones which provided molecular basis linking to neuronal excitation and epigenetic changes in ASD [86].

**Table 4:** Overview of studies using the DNSB collection

Reference	Bio-collection	Samples	Number	Study	Findings
[136]	DNSB	DBSS	1100 subjects	Chemokine analysis	Analysis of crude estimates showed decreased levels of RANTES. Adjusted estimates showed no significance amongst 3 chemokines studied (RANTES, MCP-1, MIP-1A). Cautious suggestion of altered immunity in neonatal period amongst ASD patients
[96]	DNSB	DBSS	1200 subjects	Cytokine analysis	Suggestive evidence of decreased levels of certain th-1 and th-2 like cytokines in newborns later diagnosed with ASD.
[136]	DNSB	DBSS	1029 subjects	Neurotropic factor analysis	Decreased level of neurotropic factors found in ASD patients during Neonatal period

Study numbers listed as families or subjects wherever applicable

**Table 5:** Overview of studies using the ATP/Autism BrainNet collection

Reference	Bio-collection	Samples	Number	Study	Findings
[337]	ATP and AGRE	Brain tissue, blood and lymphoblasts	18 subjects (ATP) 841 families (AGRE) 1029 families (total)	Gene expression and association analysis	Altered expression of mitochondrial genes in anterior cingulate gyrus, motor cortex and thalamus of ASD patients. Polymorphisms in *MTX2*, *NEFL* and *SLC25A27* found to be associated with ASD.
[338]	ATP	Brain tissue	18 subjects	Gene expression analysis	Reduced expression of several genes related to electron transport in anterior cingulate gyrus, motor cortex and thalamus of ASD patients
[339]	ATP	Brain tissue	57 subjects	Functional genomic study	Analysis of CNVs showed differences of what pathways are altered between children and adults; cell number, cortical patterning and differentiation in the former, and signalling and repair pathways in the latter. Prefrontal cortex samples were used
[106]	ATP	Brain tissue	33 subjects	GWAS	Patients with ASD had more genes that were up- or down-regulated in an- individual specific manner when prefrontal cortex tissue was examined
[340]	ATP	Brain tissue	126 subjects (total) 42 subjects (ATP)	Sequencing study	Recurrent deleterious mutations found in *ARID1B*, *SCN1A*, *SCN2A* and *SETD2*. Higher proportion of mutations that are deleterious, protein-altering or cause loss-of-function in ASD patients compared to controls. Cortical and cerebellar tissue was used.
[107]	ATP	Brain tissue	25 subjects	Deep sequencing study	Altered adenosine to inosine editing found in cerebella tissue from ASD patients. Dysfunctional for of editing enzyme *ADARB1* more frequently in ASD Cerebella
[341]	ATP	Brain tissue	28 subjects (ATP) 43 subjects (total)	Gene expression analysis	Signalling partners of *FMRP* and *GRM5* (*HOMER1, APP, RAC1, STEP*) shown to have altered expression in the cerebellar vermis and superior frontal cortex in ASD patients compared to controls.
[342]	ATP	Brain tissue	19 subjects	mRNA analysis	Reduction of multiple GABA receptor subtypes (A6, B2, D, E, G2, T and P2) detected in cerebella vermis and superior frontal cortex ASD patients
[343]	ATP	Brain tissue	25 subjects	Assay study	Imbalance in isoforms of precursor BDNF protein found in fusiform gryrus of ASD patients
[103]	ATP	Brain tissue	18 subjects	Transcriptional and epigenetic association analysis	Downregulation of genes related to oxidative phosphorylation and protein translation. Associations were found between specific behaviour domains of ASD and gene expression modules related to myelination, immune response and purinergic signalling. Cerebral and Brodmann area 19 tissue was used
[108]	ATP	Brain tissue	16 subjects	Methylation study	Increased methylation was found for the gene *OTXR* in ASD patients in blood and DNA from the temporal cortex
[104]	ATP	Brain tissue	107 subjects	Transcriptome analysis	Dysregulated gene expression associated with glial cells shown to have negative correlation with gene expression relating to synaptic transmission in ASD patients when Brodmann areas 10, 19 and 44 were analysed
[344]	ATP	Brain tissue	32 subjects	Transcription analysis	RORA may have dimorphic effects on gene expression in certain areas of cortical tissue between genders, and deficiency appears to cause greater gene dysregulation amongst males in both mice and humans
[345]	ATP	Brain tissue	30 subjects	Transcription analysis	*RPP25* expression is decreased in the prefrontal cortex of ASD patients
[116]	ATP	Brain tissue	23 subjects	Alternate splicing analysis and discovery	A conserved group of microexons involved in modulation of interaction domains of proteins and neurogenesis is disrupted in patients with ASD
[29]	ATP	Brain tissue	17 subjects	Methylation study	*UBE3* implicated as a contributing gene to autism and Angelman syndrome
[346]	ATP	Brain tissue	20 subjects	Anti-sense RNA study	Discovery of anti-sense non-coding RNA that binds to moesin at 5p14.1 in ASD cerebral cortex tissue
[109]	ATP	Brain tissue	40 subjects	Methylation study	4 differentially methylated regions; 3 in temporal cortex and 1 in cerebellum. 3/4 regions were again found in different samples and brain regions.
[117]	ATP	Brain tissue and lymphoblasts	36 subjects (total)	Transcription and alternative splicing study	Accelerated decrease of MS gene transcription across ageing found in ASD patient cerebral cortex samples
[347]	ATP	Brain tissue	73 subjects	Methylation study	Correlation found between reduced expression of *MECP2* and increased methylation on the promoter region
[110]	ATP	Brain tissue	24 subjects	Methylation study	Hypomethylation of mir142 and upregulation of mi-RNAs targeting *OXTR* gene in prefrontal cortex of ASD brains
[348]	ATP	Brain tissue	24 subjects	Signal transduction study	Downregulation of PI3K-Akt genes observed in fusiform gyrus tissue of ASD patients. Similar effects noted in rat brain tissue exposed to valproic acid
[349]	ATP	Brain tissue (ATP) neuronal cells	6 subjects	CHiP study	*RORA* found to regulate *2BP1*, *CYP19A1*, *HSD17B10*, *ITPR1*, *NLGN1* and *NTRK2* via transcription. Low levels of RORA causes dysregulation of these genes and associated pathways. Prefrontal cortex and cerebellum tissue was used.
[350]	ATP	Brain tissue (ATP)	153 families (other) 54 subjects (ATP)	Functional characterisation study	Variant of the HTR2A gene rs6311 in ASD patients has lower level of expression and contains extended 5′untranslated region. Speculation that this variant could be a risk factor in ASD. Frontopolar cortex tissue was used.
[351]	ATP	Brain tissue	28 subjects	Micro-RNA study	Difference in pattern of micro-RNA expression between ASD superior temporal gyrus samples and controls. Further evidence that *Mir-320*, *Mir-132* and *Mir-302* are involved in ASD.
[113]	ATP	Brain tissue	94 subjects (total) 51 subjects (ATP)	Acetylome study	Common acetylome signatures found amongst 68% of ASD cases in 5000 regulatory regions in the prefrontal and temporal cortex. Acetylome profiles were not affected by SNPs at these regulatory regions.
[352]	ATP and AGRE	Brain tissue, blood and lymphoblasts	21 subjects (ATP) 252 families (AGRE)	Association study	Variants of *LMX1B* show modest association with ASD. Analysis of mRNA from anterior cingulate gyrus is much lower in ASD patients compared to controls.
[105]	ATP	Brain tissue	36 subjects	Gene co-expression network analysis	Transcriptional and splicing dysfunction implicated in disorder. Enrichment for genes in glial, immune and neuronal modules. Gene *A2BP1* linked to alterations in splicing. Studies based on using temporal cortex, frontal cortex and cerebellum
[353]	ATP	Brain tissue	28 subjects (total) 8 subjects (ATP)	Gene expression analysis	Genes expressed at higher levels in males enriched in upregulated genes in post-mortem neocortical tissue in ASD patients, including astrocyte and microglia markers
[118]	ATP	Brain tissue (ATP) and placenta	12 subjects (ATP) 64 subjects (total)	Vitamin B12 study	Reduced levels of B12 found in ASD, aged and Schizophrenic patients compared to controls. oxidative stress found in ASD and Schizophrenia patients. Frontal cortex tissue was used
[114]	ATP	Brain tissue	98 subjects	Methylation study	Altered methylation patterns discovered in *SHANK3* gene in cerebella tissue of ASD patients
[115]	ATP	Brain tissue	20 subjects	Epigenetic study	Enrichment of 5-hmc in cerebella tissue may be associated with increased binding by *MECP2* to *RELN* and *GAD1* promotors

Study numbers listed as families or subjects wherever applicable

**Table 6:** Overview of studies using Multipe collections

Reference	Biobank	Sample type	Number	Study	Findings
[119]	SSC and AGRE	Blood and genomic data	8044 subjects (AGRE) 4348 subjects (SSC)	Genome-wide survey on translational effects	Investigation of maternal genetic effects in ASD. Validation using other data sets (SSC and AGRE) did not reproduce similar results).
[354]	SSC and AGRE	Genomic and clinical data	Subjects (AGRE) 941 1048 subjects (SSC)	Gene association study	*ATP2C2* and *MRPL19* found to be associated with language impairment and dyslexia, respectively
[122]	SSC and AGRE	Blood	AGRE–127 subjects SSC–75 subjects	Parallel sequencing study	Rare variants of the *AFF2* gene found to associated with ASD susceptibility in males
[355]	SSC and AGRE	Genomic data	359 subjects (AGRE) SS–885 subjects	GWAS	Female protective effect in ASD is not mediated by a single genetic locus.
[356]	AGRE and SSC	Genomic data	13 subjects (AGRE) 3 subjects (SSC)	WES	Loss of *CTNND2* function linked to severe ASD
[86]	SSC and TASC	Blood and lymphoblasts	15480 (total) 2475(SSC) 601(TASC)	WES	107 genes implicated in ASD. These genes are responsible for synaptic formation, chromatin remodelling and transcriptional regulation
[123]	SSC and AGRE	Blood and lymphoblasts, genomic and clinical data	5657 subjects (total) 1555 subjects (AGRE) 872 subjects (SSC)	WES	Mutations in *SHANK1*, 2 and 3 accounts for 1 in 50 ASD cases. *SHANK1* mutations linked to mild effects, *SHANK2* for moderate and *SHANK2* for severe.

Study numbers listed as families or subjects wherever applicable

Multiple bio-collections were employed to investigate *SHANK1, 2* and *3*, which are scaffolding proteins implicated in ASD. They devised a genetic screen and meta-analysis on patients and controls including cohorts from the AGRE, SSC and Swedish twin registry. In total, ~1% of all patients in the study had a mutation in this group of genes. The mutations in *SHANK3* had the highest frequency (0.69%) in patients with ASD and profound intellectual disability. *SHANK1* (0.04%) and *SHANK2* (0.17%) mutations occurred less frequently and were present in individuals with ASD and normal IQ, and ASD with moderate intellectual disability [123].

Recently Autism, Speaks, in coordination with Google and Genome Canada, have launched another initiative; MSSNG (https://www.mss.ng/). The objective of the MSSNG project is whole genome sequencing of 10000 genomes of families affected by ASD. This incorporates AGRE along with other bio-collections to sequence the entire genomes of families with autistic children, and as of the summer of 2016, it has reached the halfway goal of 5000 genomes out of 10000, with the contribution of the AGRE (1746) and TASC (458). Two studies have been published from this initiative. In the first study, genomes from 200 families were sequenced [124]. The findings revealed many of the de novo mutations (75%) from fathers, which increased dramatically with paternal age. Clustered de novo mutations however were mostly maternal origin, and located near CNV regions subject to high mutation. The ASD genomes were enriched with damaging de novo mutations, of which 15.6% were non-coding and 22.5% genic non-coding, respectively. Many of the mutations affected regulatory regions that are targeted by DNase 1 or involved in exon skipping [124]. The second study [125] featured 5205 sequenced genomes with clinical data, where an average of 73.8 de novo single nucleotide variants and 12.6 insertions/deletions/CNVs were detected per ASD patient. Eighteen new genes were also discovered (*CIC, CNOT3, DIP2C, MED13, PAX5, PHF3, SMARCC2, SRSF11, UBN2, DYNC1H1, AGAP2, ADCY3, CLASP1, MYO5A, TAF6, PCDH11X, KIAA2022* and *FAM47A*) that were not reported in ASD previously. These data clearly demonstrate that ASD is associated with multiple risk factors, and within an ASD individual, and multiple genetic alterations may be present. The Whole genome sequencing is therefore a powerful tool to detect genetic changes at all levels. Resources like MSSNG are valuable, and pooling of ASD bio-collections are essential for identification of the common and subgroup-specific pathways and drug targets of such a multi-factorial disease of ASD which involves hundreds of risk factors.

### Stem cell research and autism spectrum stem cell resource

A major impediment to recent drug discovery particularly in the field of neuroscience is the lack of human cell models. The iPSC technology developed by Nobel Laureate Shinya Yamanaka has provided an excellent opportunity [126]. Fibroblasts from patients’ biopsy can be converted into iPSCs with defined transcription factors, which resemble embryonic stem cells and can become most cell types in our body. Therefore, patient-derived iPSCs may be used to investigate disease pathology, progression and mechanisms to create human disease models for drug screening and testing [127, 128].

The SSC has also commenced efforts to create iPSC lines from idiopathic ASD patients who have large head circumference but unknown gene association [129]. The iPSCs were grown into organoids to mimic cortical development, and ASD organoids were shown to display a disproportionate ratio of inhibitory: excitatory neurons. The cortical gene *FOXG1* was overexpressed in ASD organoids, and this overexpression correlated with the severity of ASD and their head size [129]. This study has demonstrated a proof-of-concept to model ASD in culture stem cells.

The Children’s Hospital in Orange County California has set up a bio-collection dedicated to this task, the ASD Stem Cell Resource. ASD patients were screened and accepted based on the following criteria: ASD patients if they have no other conditions (i.e. trauma, stroke, seizure disorders) affecting the central nervous system other than ASD; if they have no features of other known genetic conditions (e.g. tuberous sclerosis); Fragile X patients if they are genotypically confirmed for the CGG repeat number of the *FMR1* mutation; idiopathic autism patients who are negative for *FMR1* mutation and chromosomal abnormality; if they possess an IQ of 40 or greater, and if they are 8-year-old or above. Skin punches and blood were collected in one location (MIND Institute), and fibroblasts were cultured and stored at the Hospital. The collection has been organised into seven groups; unaffected controls, Fragile X without ASD, Fragile X with ASD, permutations without ASD, permutations with ASD, ASD (not meeting full criteria for idiopathic status) and idiopathic ASD.

As of 2014, this resource was composed of iPSCs from 200 unaffected donors and patients. The collection includes fibroblasts, blood, iPSCs, iPSC-derived neuronal and glial cells. The first study published using this bio-collection was the iPSC models of Fragile X syndrome [130]. The Fragile X patient fibroblasts were used to derive iPSCs and differentiate into neurons for transcriptomic analysis. The neuronal differentiation genes (*WNT1, BMP4, POU3F4, TFAP2C*, *PAX3*) were shown to be upregulated, whereas potassium channel genes (*KCNA1, KCNC3, KCNG2, KCNIP4, KCNJ3, KCNK9*, *KCNT1*) were downregulated in Fragile X iPSC-derived neurons. The temporal regulation of *SHANK1* and *NNAT* genes were also altered, with reduced *SHANK1* mRNA and increased *NNAT* mRNA in patient cells. While the stem cell collection is relatively new, it has great potential to facilitate brain cell culture in vitro, which would otherwise not be feasible by using post mortem brain tissue.

## Discussion

It is clear from the studies reviewed here that large ASD bio-collections have had an undisputable impact on progressing genomic discovery in ASD, leading to enhanced understanding of ASD neurobiology. While many studies used private collections as sources for tissue and data, large and well characterised samples from the collections reviewed have supported the discovery of small genetic effects, e.g. in GWAS and rare genetic mutations such as pathogenic CNV and SNV but it is clear, as highlighted for other neurodevelopmental disorders such as Schizophrenia that larger samples are required. Both genetic and phenotypic heterogeneity are impediments to gene discovery. Large bio-collections aim to reduce these effects but challenges remain. Each of the bio-collections reviewed has its own strengths and limitations.

### Phenotypic and genotypic heterogeneity

Some of the bio-collections, e.g. SSC, AGRE, TASC, reduced phenotypic heterogeneity through the use of research gold standards for ASD diagnosis, ADI-R and ADOS. Different versions of these instruments based on the timeline when these data have been collected have been used. IQ measurement is more complex to calculate due to the broad range of IQ commonly included within bio-collections. Differences also exist in the clinical profile of subjects included in the different collections with some samples, e.g. SSC, comprised of more individuals with higher cognitive functioning relative to AGRE, TASC or AIC. Medical and psychiatric comorbidities [7] have greater recognition but are not as systematically evaluated in each of the collections. Differences in ascertainment are also relevant. The SSC focused on simplex autism, i.e. families where only one child was affected to maximise the detection of rare variants. Consequently, the relative contribution of common genetic risk within the SSC sample appears reduced. In contrast to autism specific bio-collections, the DNSB, provides a large population-based sample with clinical diagnosis that can maximise power within GWAS studies to detect common genetic variation but does not provide in-depth clinical data for phenotype-genotype analyses. This was evident in the studies on amniotic fluid and DBSS where different diagnostic criteria would have been applied at the time of the subjects’ diagnoses, meaning one criteria would have excluded subjects(ICD8) whereas another would not (ICD10) [95, 96] [93, 94].

Throughout the studies listed here, there is an imbalance of ethnicities of bio-collections, as many of the studies rely heavily on Caucasian/European descent, which has been pointed out in some journals [131] and should consider diverse family structures [132], which can otherwise lead to population stratification [133]. Fortunately, efforts are underway to explore genetics of ASD in other countries such as China [134] and Brazil [135], which will reinforce many of the earlier findings covered in this review.

### Samples

Large collections providing DNA for genomics studies have been advantageous; however, as studies move beyond the scope of genetics into transcriptomics, epigenomics and proteomics, a wider variety of sample types will be required. Serum will be valuable for investigating circulating metabolites and proteins that are expressed peripherally, including chemokines [93, 95], cytokines [94, 96], neurotropins [136], MMPs [137] and hormones [138]; however, this may not be the most appropriate tissue to investigate brain relevant ASD genes and proteins. DBSS, which can be useful for WES [139, 140], methylation [141] and gene expression [142], would not be as useful as fresh drawn blood for WGS, as DBSS-derived DNA would need to be amplified prior to use for analysis, potentially causing bias.

However, human brain tissue is a rare resource; brain tissue is very difficult to access due to its scarcity, and the preservation methods used may limit studies being carried out. Also, the types of brain cells are dependent on brain tissue being used; neuronal tissue in grey matter or glial tissue in white matter. Many of the studies listed in the Autism BrainNet, for example, utilised certain sections of the brain; and the most commonly used sections are the prefrontal cortex, temporal cortex, Brodmann’s area, cerebellum and cingulate gyrus. While findings from these sections have been of crucial importance, a capacity to model the entire brain and to observe progression of ASD development would be ideal, and patient’s somatic cells can now be converted to iPSCs and then into disease cell types.

IPSCs have been used as disease models for Fragile X syndrome [143–145] and Rett syndrome [146], and iPSCs have been generated from patents with deletions in *SHANK3* [147] which are implicated in a number of neurodevelopmental disorders. The three-dimensional culture is developed and iPSCs can also be used to create mini-organoids, which can come very close to mimicking aspects of brain development [129, 148]. In addition to the brain cell types discussed earlier [129, 149], the iPSCs could be used to generate other cell types implicated in ASD co-morbidities, such as the gut [88, 150] and the blood brain barrier [151, 152].

Fibroblasts are the first cell type used to make iPSCs from mice [126] and humans [153] and remain as the most popular cell type for generating neural stem cells, neurons or iPSCs. Fibroblasts are easier to reprogram than many other somatic cells, and the reprogramming efficiency is between 0.1–1% depending on the reprogramming method [154]. They require basic culture media and proliferate rapidly, so large numbers of fibroblasts can be generated in a short period. Unlike keratinocytes they require trained medical personnel to obtain skin biopsies, which could be distressing to some ASD patients. Low passages of fibroblasts are required for reprogramming as higher passages dramatically reduce reprogramming efficiency and increase genomic instability [155].In addition to their use for IPSCs, fibroblasts can be used to investigate amino acid transport, and ASD fibroblasts were found to have greater affinity for transporting alanine, but less affinity for tyrosine—a key component for the synthesis of the neurotransmitter dopamine [156]. Fibroblasts can be used as a proxy to investigate transport across the blood-brain barrier [156, 157] and to investigate calcium signalling [158, 159].

Keratinocytes can also be used for generating IPSCs [160]. Collection is less invasive than skin biopsy and can be carried out by non-medical personnel. The hair samples are easy to transport and culture and transformed cells are easier to identify and isolate. Similar to fibroblasts, keratinocytes are reprogrammed at low passages and fewer methods have been employed to reprogram keratinocytes than fibroblasts. The lentiviral, retroviral and episomal reprogramming were tried successfully [155, 161, 162], and keratinocytes were shown to have high reprogramming efficiency of 1–2%. The major challenge is the reproducibility of keratinocyte growth, and it often requires repeated rounds of hair plucking from a same donor.

### Organization

There are many generic articles and white papers for biobanks available, including consensus best-practice recommendations. For those who may wish to start their own bio-collections, we have listed a few articles in Table 7 for further reading on topics pertaining to collection, management, sustainability and quality control. In addition, links to international guidelines can be found here (http://www.oecd.org/sti/biotech/guidelinesforhumanbiobanksandgeneticresearchdatabaseshbgrds.htm; http://www.isber.org/?page=BPR; https://biospecimens.cancer.gov/practices/). However, even when using best practice guidelines, the storage and use of bio samples will be subject to the laws where the facilities are located, and will vary from country to country [163].

**Table 7:** Description of papers relating to aspects of biobanking

Reference	Subject of paper
[357]	Introduces concept of adding value to stakeholders (patient donors/funders/research customers) and to find balance between aspects of sustainability (acceptability/efficiency/accomplishment)
[358]	Feasibility of simplified consent form for biobanking. Result indicates simplified forms combined with supplemental information for further reading effective in minimising form length and complexity
[359]	Review paper detailing best practice guidelines for sample collection and storage, management of data and infrastructure. In addition, ethical, legal and social issues are explored
[360]	Paper discussing aspects of embryonic stem cell banking that can be applied to iPSCS
[361]	Key issues relating to delivery and safety testing of iPSC stocks for use in research and therapy. Importance of international and national coordinated banking systems are also discussed
[362]	Description of enclosed culture system for iPSCS and neural precursors for use in preclinical and basic research

### Participation and ethics

Stakeholders can have a considerable influence on how a bio-collection operates and how a bio-collection can be set up, managed and monitored [164]. In addition to researchers, clinicians and parents in bio-collections of ASD research, autistic stakeholders should be included as part of the stakeholder group, which could help guide and inform how research is carried out. A recent survey [165] was carried out amongst researcher-community engagement on ASD research in the UK. A high dissatisfaction and level of disengagement was expressed by parents and patients, who felt that research outcomes made little or no difference to their day-to-day lives and that they were not communicated, not involved or valued. Patients also felt that they did not receive follow-up and researchers were unapproachable and driven by data collection. Establishment and sustainability of a good stakeholder engagement are essential in ASD research and in biobanking. This will not only help guide research to subjects that matter to the community, but also the future of the biobank. One initiative, such as SPARK (Simons Foundation Powering Autism Research for Knowledge) is underway to encourage ASD communities in the USA to participate in ASD research. While such a goal is laudable, it is crucial that participants are engaged in the entire process. They are not just the suppliers of bio-collections for research and data collection, but also make an input into research areas, which directly impinge on the quality of their life. Meanwhile, regular public events to update research progress and challenges to the stakeholder community may help win their understanding, appreciation and continuous support.

The ethics and obtainment of consent are significant factors for bio-collection research. The main considerations include what information shall be given to potential donors regarding the protocol and its implications of the research, how consent should be obtained [166] or what shall be done if consent was not clearly given [167]. It is also a matter of debate whether the consent should be “broad” and if the patient shall consent to a framework of research; if ethical review of each project shall be carried out by independent committees, and what are the strategies to inform and renew consent if there is significant deviation of framework; where shall the consent be revisited and renewed for every new study [168]; how the data will be protected and accessed [169, 170]; and how the findings will be communicated [171]. The latter is especially important if findings are of clinical significance to certain donors or it may affect their health or well-being [167]. These are the issues that each ethical application faces in making the application.

For people with ASD, it can be very complicated. Parents will give consent for their children if they want to donate samples for the bio-collection, but there is a question of adults who may not have the ability to give consent or to fully understand the implications. It is also important to clearly communicate what this research will mean for the patient and the family, including findings that may be of pathological as well as clinical significance. Liu and Scott have commented on how the discoveries made in ASD research can be distorted by media. If parents/patients are misled to believe that a cure will come out a few years down the road, this may lead to disappointment and make them reluctant to participate in further research. Liu and Scott pointed out that the Neurodiversity Movement group (high-functioning autists) would have issues with certain research. They will not participate in research if they feel it may threaten or undermine people with ASD [128]. They prefer investment on services and therapies, rather than on genetic studies which may result in prevention of autistics being born [172–174], and the idea of curing autism is a complicated topic of debate [175].

For iPSC research, it was suggested to educate participants on the current state of research, to clearly explain the benefits and risks of biopsy donation and to consult the ASD community on research focus of an ASD bio-collection and on distribution of the cell lines [128]. For clinical trials of stem cells, stem cell counsellors shall inform participants the benefits and risks of enrolling in stem cell trials and to safeguard them from the dangers of stem cell tourism. Such an approach should also be considered for ASD-related studies [176].

## Conclusions

In conclusion, bio-collections have been shown as valuable resources and enabled large-scale studies on ASD. The recent genetic studies have begun to reveal de novo mutations on major cellular pathways [17, 177]. There is also emerging evidence that ASD continuum contains subgroups with discrete mutations in specific genes such as *CDH8* [88], *DYRK1A* [71] and POGZ [90] and gene mutations like *NRXN1* [28, 60, 73, 178, 179] and *SHANKs* [72, 98, 114, 123] recurring in broad populations. There is a vast amount of clinical and biological information available in these bio-collections, and the data are in the need for concrete guidelines on ethics and governance. The communication and trust shall be maintained between the researchers and families who have given biological and personal information. Finally, the availability of iPSC resources dedicated to idiopathic and syndromic forms of ASD could be a tremendous boon to the research community and such models are anticipated to be complementary with animal models and to speed up the development of therapeutic interventions for ASD. They could open up the possibilities of functional studies of ASD on a large scale and could become a future model for other iPSC bio-collections to be set up worldwide.

## Abbreviations

ADI-R: Autism Diagnostic Interview–Revised
ADOS: Autistic Diagnostic Observation Schedule
AGP: Autism Genome Project
AGRE: Autism Genetic Resource Exchange
AIC: Autism Inpatient Collection
ASD: Autism spectrum disorders
ATP: Autism tissue program
CAN: Cure Autism Now Foundation
CCGSMD: Centre for Collaborative Genetic Studies on Mental Disorders
CNV: Copy number variation
DBC: Danish Birth Cohort
DBSS: Dried blood spot samples
DNSB: Danish Newborn Screening Biobank
DSM-5: Diagnostic and Statistical Manual of Mental Disorders
GSH: Glutathione
GWAS: Genome-wide association study
HAWAS: Histone acetylome-wide association study
HBDI: Human Biological Data Interchange
ICD: International Statistical Classification of Diseases and Related Health Problems
iPSC: Induced pluripotent stem cells
MMP: Matrix metalloproteinase
MPLA: Multiplex ligation-dependent probe amplification
PDD-NOS: Pervasive developmental disorder not otherwise specified
RUCDR: Rutgers University Cell and DNA Repository
SCD: Social (pragmatic) communication disorder
SNP: Single nucleotide polymorphism
SNV: Single nucleotide variation
SSC: Simons Simplex Collection
TASC: Autism Simplex Collection
WES: Whole exome sequencing study
